# Whole body vibration therapy: a novel potential treatment for type 2 diabetes mellitus

**DOI:** 10.1186/s40064-015-1373-0

**Published:** 2015-10-06

**Authors:** Hongyu Yin, Henrik O. Berdel, David Moore, Franklin Davis, Jun Liu, Mahmood Mozaffari, Jack C. Yu, Babak Baban

**Affiliations:** Department of Oral Biology, Georgia Regents University, Building: CL 2140, 1120 15th Street, Augusta, GA 30912 USA; Plastic Surgery Hospital, Chinese Academy of Medical Science and Peking Union Medical College, Peking, China; Palmetto Health/University of South Carolina School of Medicine, Columbia, SC USA

## Abstract

There is a worsening epidemic of obesity and diabetes in the world. Life style interventions including dietary changes and increase in exercise can improve glucose metabolism and health in general. However, standard exercise programs are strenuous, time-consuming, and thus have low long-term compliance issues. We tested the feasibility of using high frequency, low amplitude whole body vibration (WBV) therapy to improve glucose metabolism in young type 2 diabetic (T2DM) mice. We also aimed to investigate the postulated anti-inflammatory and cytoprotective properties of WBV. Male db/db and db/m mice were exposed to high frequency, low-amplitude WBV. Outcome parameters comprised of body weight, hemoglobin A1c (HbA1c) level, as well as interleukin (IL)-17 (a marker of helper T cells), forkhead box P3 (Foxp3; a marker of regulatory T cells), and gammaH2AX (an index of DNA injury) expression. Furthermore, a 24 h metabolic cage study was carried out immediately after the WBV protocol and fluid intake, urine excretion and urine osmolality were determined. WBV did not affect body weight but improved HbA1c levels in db/db mice. Vibrated db/db mice demonstrated less fluid intake and urine excretion but better urinary concentrating ability than their non-vibrated controls. Pro-inflammatory changes were significantly reduced, as indicated by reduced IL-17 but increased Foxp3 expression. WBV reduced gammaH2AX in db/db mice suggestive of cytoprotective effect. However, WBV was largely without significant effects on assessed parameters in db/m mice. Collectively, our findings suggest that daily, short duration WBV may improve glycemic control, polydipsia, polyuria, and urine osmolality in T2DM in association with reduced inflammation. Thus, WBV may be a viable adjunctive treatment strategy in T2DM.

## Background

An unintended but inevitable consequence of decrease in physical labor and increase in productivity in centuries following the industrial revolution is the progressive uncoupling between caloric intake and energy expenditure, with the former exceeding the later. The trend of excess caloric intake and reduction in food variety accelerated in the latter part of the twentieth century. This phenomenon combined with a progressively more sedentary life style was predicted in the 1980s to create a perfect storm of explosive rise in the so called Lifestyle-Related Chronic Diseases such as hypertension, diabetes, atherosclerotic cardiovascular diseases and obesity (Eaton et al. 1988). Diabetes is already the fifth-leading cause of death in the US, with mortality rates increasing by 45 % since 1987. From 1980 to 2010, the prevalence of diabetes has increased by 300 %, according to data from the Center for Disease Control and Prevention (Roger et al. 2011). Further, according to the National Diabetes Statistics Report (2014), an estimated 29.1 million American are afflicted with diabetes mellitus, 8.1 million of whom are undiagnosed; the vast majority of these individuals have type 2 diabetes mellitus (T2DM).

T2DM is characterized by excessive hepatic glucose release, central obesity, impaired pancreatic insulin secretion and decreased insulin sensitivity by target cells leading to insulin resistance with chronic and persistent hyperglycemia. Peripheral insulin resistance occurs because of impaired insulin-induced signal transduction that normally causes membrane translocation of glucose transporters such as GLUT4 from the cytosol (Hughes et al. 1993). GLUT4 is specific to skeletal muscle cells and adipocytes, facilitating insulin stimulated movement of glucose into the cell.

Many studies have shown that exercise improves glycemic control in patients with T2DM (Baum et al. 2007; Davis and Holm 2012). This beneficial effect is likely multifactorial, including increasing energy expenditure as well as insulin-induced membrane translocation of GLUT4. In addition, a number of studies have shown that exercise improves glycemic control in T2DM patients and in animal models, in part, due to anti-inflammatory properties (Teixeira-Lemos et al. 2011; de Lemos et al. 2007; Teixeira de Lemos et al. 2009). However, sustained exercise routine is strenuous, time consuming, and difficult to maintain given today’s rapid paced digital society (Unick et al. 2010). There is a great need to increase the efficiency and compliance of conventional exercises, achieving the euglycemic and other health benefits of exercise without the vigor and duration of standard exercise intervention programs (Brownell 1986). Because exercises and whole body vibrations both exert mechanical stresses on the skeleton, whole body vibration (WBV) therapy may be one answer to such exercise modifications. We investigated the effects of standard high frequency, low-amplitude synchronous vertical WBV delivered by a commercially available platform in young adult db/db mice, a model of T2DM, with the objective of answering the following questions:

1. Is there an effect of WBV on body mass?
2. Can WBV reduce HbA1c in diabetic mice?
3. What is the effect of WBV on indices of kidney function (a major target of T2DM)?
4. Can WBV improve circulating inflammatory profile and reduce cell damage?

## Methods

### Animals

Eight-week-old male db/m and db/db mice were obtained from the Jackson Laboratories (Boston, MA, USA) and housed in the laboratory animal facilities at the Georgia Health Sciences University. The use of animals for these studies were in accordance with ethical standards and was approved by the Institutional Animal Care and Use Committee.

### Vibrating design and fabrication

**Fig. 1:**
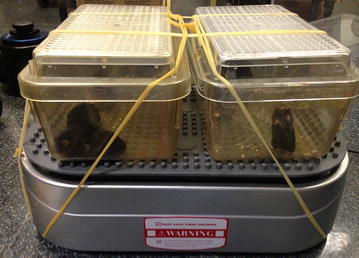
Picture of vibrating platform capable of delivering the prescribed high frequency, low amplitude vibration (“Methods”)

To deliver synchronous vertical whole body vibration (WBV) with controlled amplitude and frequency, we used a commercially available standard WBV machine offering 12 speeds (Vibratone AB-760, Hacienda Heights, California 91745, USA). We selected speed 1 (frequency: 30 Hz; amplitude: 3 mm) for 20 min/day for 5 days per week for 6 weeks. The amplitude is the maximum displacement distance of the platform surface on which the mice were placed. The frequency of 30 Hz and magnitude of 0.5–1.5× gravitation (4.9–14.7 m/s^2^) were verified by accelerometer. Figure 1 shows a picture of the custom-designed vibrating platform used in this study.

### Effects of WBV in diabetic and non-diabetic mice

To test the effects of WBV, four groups of male mice (i.e., db/db mice, a model for T2DM, and their non-diabetic non-obese db/m controls) were used at 2 months of age (n = 6 animals/group): non-vibrated db/db; vibrated db/db; non-vibrated db/m; vibrated db/m. After 6 weeks, the animals were individually placed in metabolic cages to monitor water intake, urine excretion and subsequent measurement of urine osmolality. HbA1c levels were measured from blood collected at the time of sacrifice (A1CNow, Bayer HealthCare, LLC, Tarrytown, NY, USA).

### Effects of WBV in the weight of Diabetic and non-diabetic mice

To determine the effects of WBV on the body weight, experimental animals were weighed before and after commencement of WBV at one-week interval for a total of 7 times.

### Assessment of inflammation and cell death

For the assessment of parameters of interest, fluorescence-accelerated cell sorting (FACS) of whole blood was performed, using FACSCalibur flow cytometer (BD BioSciences, San Diego, CA, USA), as previously described (Baban et al. 2013a, b, c). In brief, cells were washed twice with cold phosphate-buffered saline and then resuspended in binding buffer and gently vortexed before incubation with IL-17, Foxp3, or gammaH2AX antibodies. Cells were analyzed by flow cytometry within 1 h after adding binding buffer.

### Antibodies and kits

Antibodies against the pro-inflammatory cytokine IL-17, as well as the anti-inflammatory marker Foxp3 were purchased from EBIOSCIENCE (San Diego, CA, USA), whereas gammaH2AX antibody was obtained from Cell Signaling (Denver, MA, USA).

### Statistical analysis

Data were analyzed using the analysis of variance (ANOVA) followed by Newman-Keuls post hoc test to establish significance (p < 0.05) among the groups. Further body weights were analyzed by repeated measure ANOVA. Data are expressed as mean ± SD.

## Results

### Effects of WBV on the weight of db/db and db/m mice

**Fig. 2:**
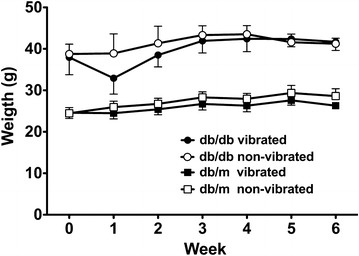
Body weight was significantly (p < 0.05) higher in db/db than db/m mice during the course of the study; data are mean ± SD of 6 animals/group

As expected, body weight of db/m mice was significantly (p < 0.05) lower than that of db/db mice during the course of the study (Fig. 2). Vibrated db/db mice displayed a reduction, albeit non-significant, in body weight after 1 week of WBV. Thereafter, however, body weight was similar between vibrated and non-vibrated db/db mice. Similar to db/db mice, WBV did not markedly affect body weight of db/m mice.

### Effects of WBV on glucose metabolism in 8-week-old db/db and db/m mice

**Fig. 3:**
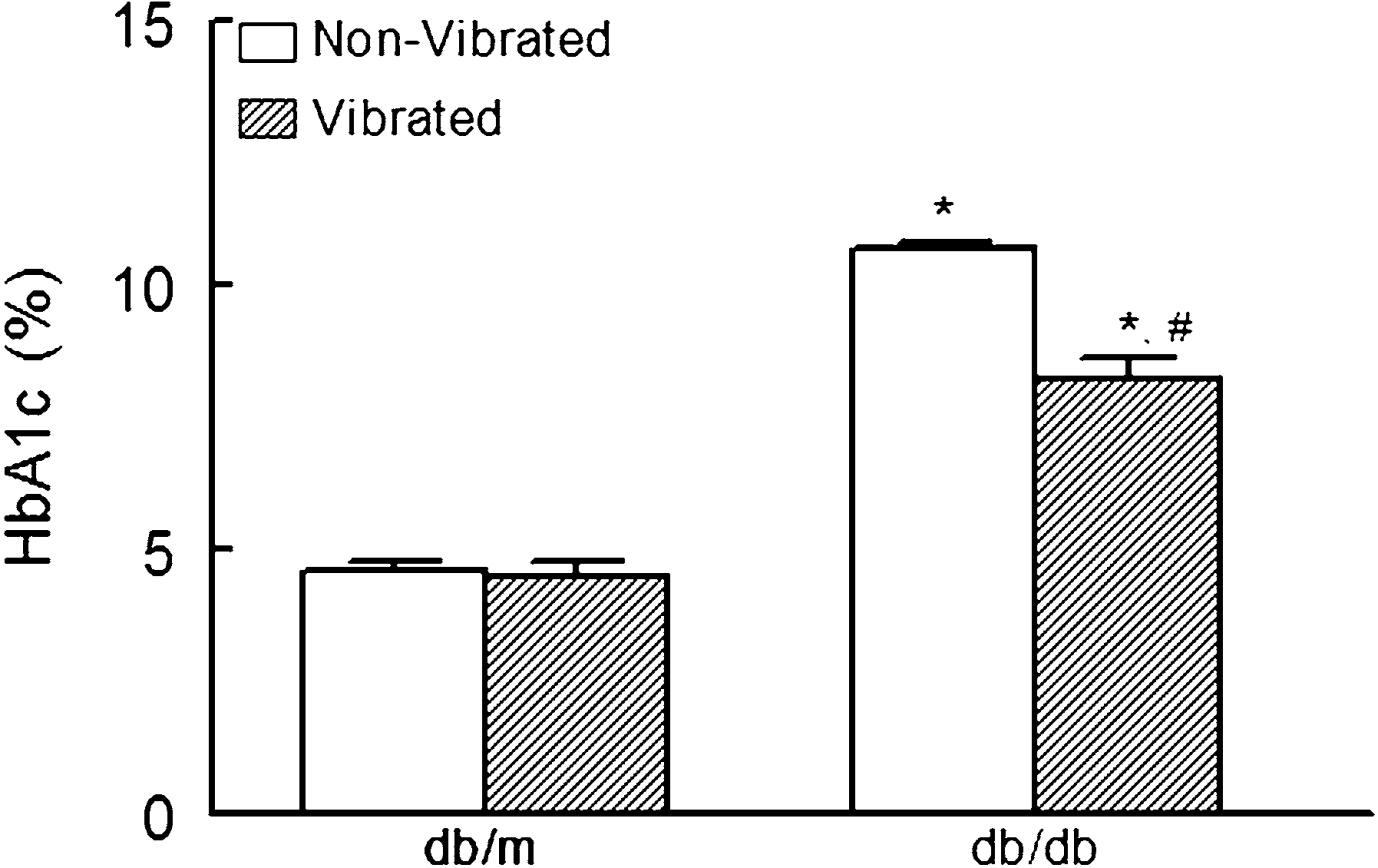
HbA1c levels of non-vibrated and vibrated db/m and db/db mice; data are mean ± SD of n = 6 animals for each group. *p < 0.05 compared to their respective db/m controls. **p < 0.05 compared to their non-vibrated counterparts

As shown in Fig. 3, HbA1c levels were significantly higher in the db/db than db/m mice. WBV reduced HbA1c (about 22 %; p < 0.05) in vibrated than non-vibrated db/db mice. On the other hand, WBV did not affect HbA1c level in db/m mice. Due to the small sample size, the effect size index was calculated based on Cohen’s *d* formula (Kenny 1987). The effect of intervention is considered large if the Effect Size Index is more than 0.8, moderate for 0.5, and small for less than 0.2. The Effect Size Index for the decrease in HbA1c in db/db mice is large (1.97), indicating a high likelihood of reproducibility.

### Effects of WBV on indices of kidney function in 8-week-old db/db and db/m mice

In the non-diabetic non-obese db/m mice, WBV increased fluid intake but it did not affect urine output or urine osmolality (Table 1). On the other hand, WBV reduced fluid intake by about 25 % in db/db mice. Vibrated db/db mice also excreted less urine. Moreover, WBV improved urinary concentrating ability as demonstrated by a 28 % increase in urine osmolality in vibrated db/db mice (Table 1). Collectively, the results indicate a prominent effect of WBV on urinary parameters in db/db, than db/m, mice as also revealed by larger effect sizes indicating a high likelihood of reproducibility (Table 1).

**Table 1:** The metabolic cage results from db/m with and without WBV; data are mean ± SD of n = 3 animals/group

	Fluid intake (mL/24 h)	Urine excretion (mL/24 h)	Urine osmolality (mOsmol/kg)
db/m Non-vibrated	1.33 ± 0.58	0.43 ± 0.06	2480.0 ± 303.2
db/m vibrated	1.00 ± 0.00^#^	0.30 ± 0.17	3628.5 ± 691.0
db/db Non-vibrated	21. 00 ± 0.00*	17.00 ± 1.00*	1084.17 ± 65.26*
db/db Vibrated	15. 67 ± 1.53*^,#^	13.33 ± 1.53*^,#^	1387.50 ± 133.30*^,#^
Size effect index; non-vibrated vs. vibrated db/m	1.94	1.07	1.59
Size effect index; non-vibrated vs. vibrated db/db	1.90	1.73	1.74

* p < 0.05 compared to their db/m counterparts

^#^p < 0.05 compared tp their non-vibrated counterparts

### Effects of WBV on inflammation and cell damage

**Fig. 4:**
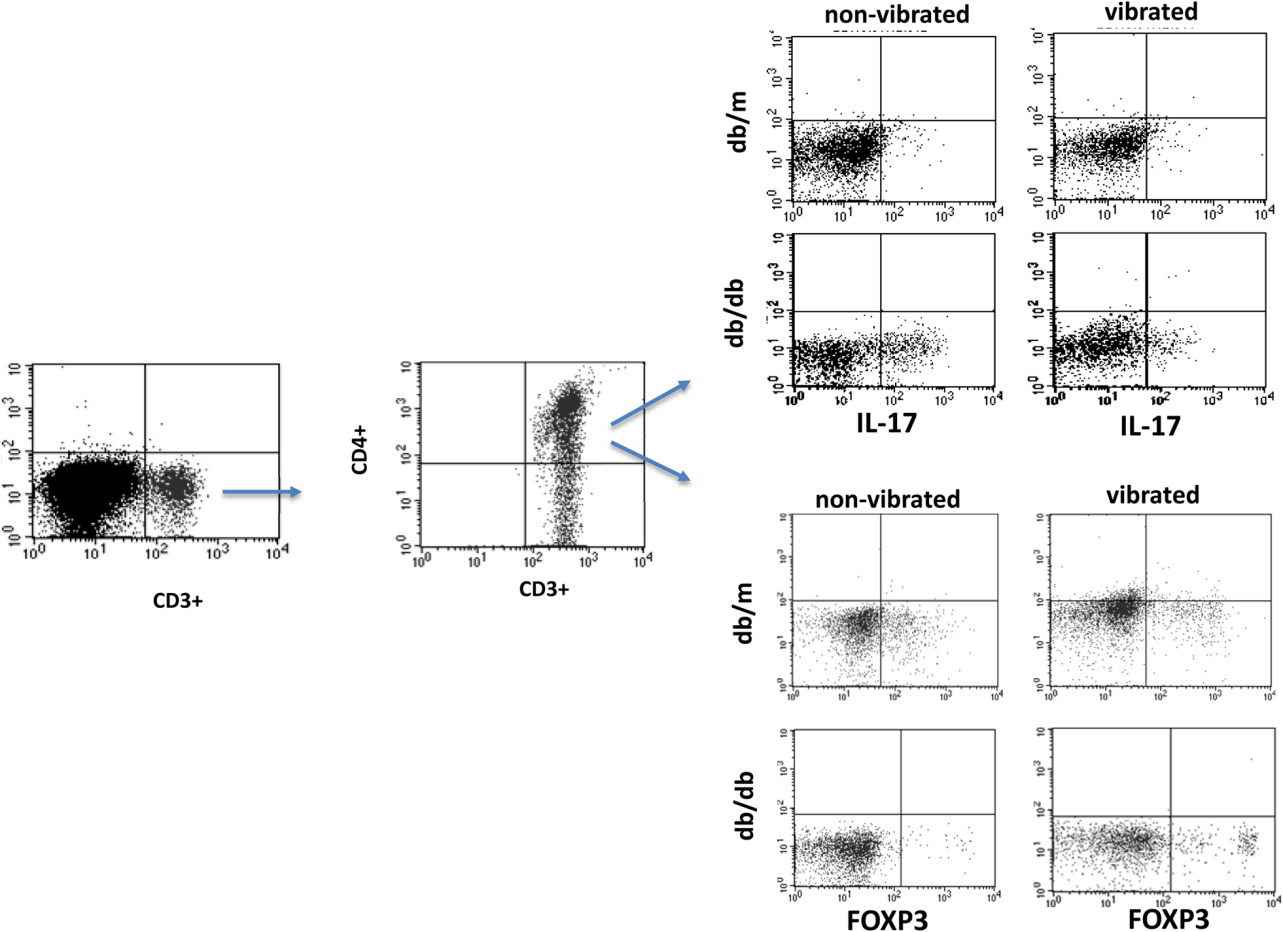
Representative FACS dot plots of IL-17 and FoxP3 positive T cells in vibrated and non-vibrated db/m and db/db mice. CD4+ T cells of peripheral blood from experimental groups were further identified as those positive for IL-17 and FoxP3

**Fig. 5:**
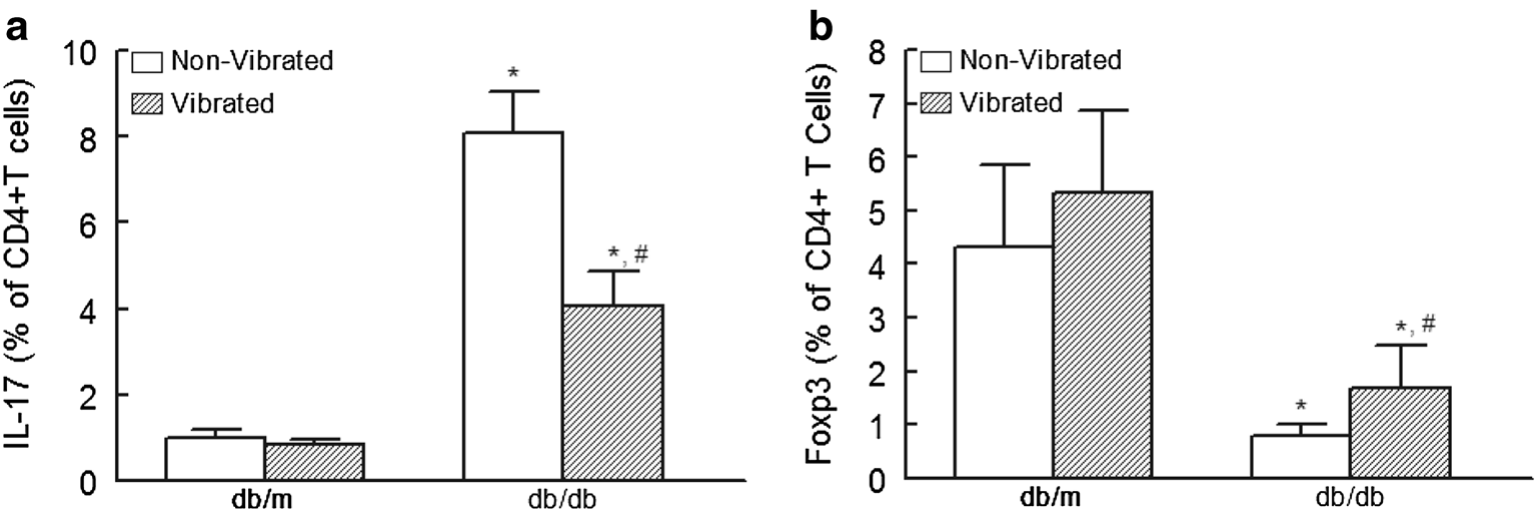
FACS analysis of IL-17 (**a**) and Foxp3 (**b**) positive cells of peripheral blood obtained from vibrated and non-vibrated db/m and db/db mice; data are mean ± SD of percent of CD4+ cells of n = 6 mice/group. *p < 0.05 compared to their respective db/m controls. **p < 0.05 compared to their non-vibrated counterparts

**Fig. 6:**
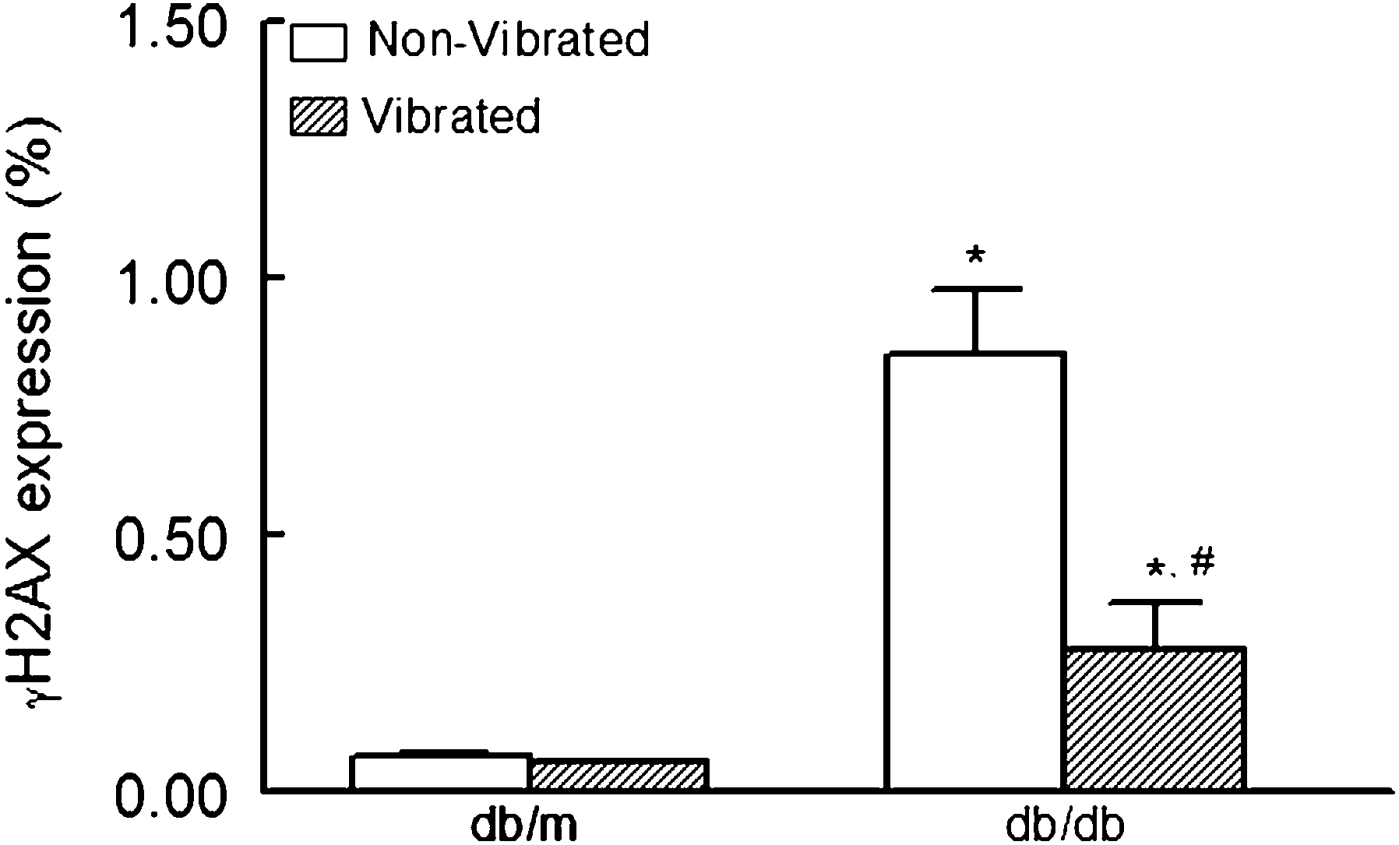
FACS analysis of γH2AX (gamma-H2AX) expression of vibrated vs. non-vibrated db/m and db/db mice; data are expressed as mean ± SD of percent of total peripheral blood cells (n = 6 animals/group). *p < 0.05 compared to their respective db/m controls. **p < 0.05 compared to their non-vibrated counterparts

In order to investigate the postulated anti-inflammatory effects of WBV, whole blood of experimental animals at the end of the experiment were analyzed for IL-17 and Foxp3 expression (Figs. 4, 5); IL-17 is a pro-inflammatory cytokine and a marker of T helper cells, whereas Foxp3 serves as an anti-inflammatory marker of regulatory T cells (Aarvak et al. 1999; Zhang and Zhao 2007). Figure 4 depicts representative flow cytometry panels, for each experimental group, identifying CD4+ cells which were subsequently identified as those positive for IL-17 and FoxP3. As shown in Fig. 5, while there is a significant difference in both IL-17 (panel a) and Foxp3 (panel b) expressing cells between db/m and db/db mice, WBV was without significant effects on these parameters in db/m mice. On the other hand, IL-17 positive cells were significantly reduced in the vibrated than non-vibrated db/db mice. Further, Foxp3 positive cells were showed a mild increase in vibrated than non-vibrated db/db mice. In order to determine whether WBV reduces cell injury, we determined expression of gammaH2AX which is a marker of DNA injury (Kuo and Yang 2008; Mozaffari et al., 2012). As shown in Fig. 6, db/db mice showed significant increase in gammaH2AX expression in db/db than db/m mice, irrespective of WBV treatment. However, the percent of whole blood cells which were positive for gammaH2AX were markedly reduced in vibrated than non-vibrated db/db mice.

## Discussion

Likely driven by global climatic changes, human energy metabolism evolved some 3–4 million years ago at a time when fat and protein intakes were sporadic, unpredictable, and very energy-requiring (Ungar et al. 2006). With the appearance of tool making during the Pliocene Epoch, the ability to hunt terrestrial mammals and to break cranial and long bones of the prey permitted the early hominins to access food sources high in polyunsaturated fats from brain and bone marrow. Because of the unpredictability and high energy expenditure of such food sources, the ability to store energy by converting excess macronutrients into adipose tissue conveyed great survival advantages for the individual (Hockett and Haws 2003). However, with the readily available high fat, high carbohydrate fast foods, and generally reduced reliance on physical vigor in today’s environment, there is a vast and persistent discrepancy between caloric intake and energy expenditure, resulting in ever increasing prevalence and incidence of obesity. Associated with this is an unrelenting rise in the incidence of many chronic diseases, including hypertension, atherosclerotic cardiovascular diseases, and T2DM (Mokdad et al. 2003). Based on large epidemiological and econometric studies, the negative impact of these chronic diseases, many of which are lifestyle-related, is very significant, consuming an estimated 75 % of the entire healthcare budget in developed countries by the early 2000s (Suhrcke et al. 2006). Even though the benefits of standard exercise intervention programs in improving serum glucose clearance have been well-documented, compliance rate is low and recidivism rate is high, because exercise is strenuous and time-consuming resulting in low compliance with such recommendation (Jakicic 2012).

During exercise, parts of the skeleton undergo cyclic strain from stresses produced by muscle contractions and gravitation. This creates fluid flow within the strain-magnifying lacuno-canalicular systems of the bone with osteocytes experiencing shear stress in the range of 0.8–3 Pa (Burger and Klein-Nulen 1999). Such cyclic shear stress in this biological microfluidic poro-visco-elastic composite is an important stimulus for osteocytes to signal osteoblasts to synthesize and release bone specific proteins including osteocalcin, a glutamate-rich protein previously thought to be a structural protein (Nomura and Takano-Yamamoto 2000). Under acidic conditions, the glutamic acid residue of a small fraction of osteocalcin escapes gamma carboxylation, and the under gamma-carboxylated osteocalcin enters the circulation to serve very important endocrine functions, including increasing insulin sensitivity and responsiveness (Prats-Puig et al. 2010). Osteocalcin knockout mice have been shown to develop central obesity, hypertension, and impaired glucose tolerance (Lee et al. 2007). Bone, in addition to being the rigid structural support for the body, is now considered an important endocrine organ (Motyl et al. 2010; Schwartz et al. 2012). Whatever the mechanism, how to modify current exercise programs to increase their effectiveness and efficiency is critically important if such modality is ever to achieve generalized acceptance and improved compliance. There are many large cross-sectional, population-based studies correlating better glycemic control and lipid metabolism in individuals with higher serum osteocalcin levels (Zhou et al. 2009). In a cross-sectional clinical study, higher osteocalcin level was highly and significantly correlated to better body mass index, insulin sensitivity as well as fasting glucose and insulin levels (Pittas et al. 2009).

WBV has been used in cosmonaut training by the Russian space program for decades and is well-adopted in European exercise centers (Rittweger 2010). In a clinical report from Germany in 2007, conducted in 40 elderly patients with T2DM, best glycemic control was found in the WBV group compared to other conventional exercises. Using daily horizontal swing type WBV at 30 Hz and 2 mm maximum amplitude for 3 weeks, the investigators found a decrease in fasting peak glucose concentration of 6.3 % using oral glucose tolerance testing (Baum et al. 2007). Vibration of the entire stationary body through both feet at frequency of 30 Hz can also occur in a vertical orientation, known as the synchronous WBV. Most protocols call for maximum vertical acceleration, A_peak_, during up stroke, of 0.5 g above the gravitational level of 9.8–14.7 m/s^2^, and reduction by 0.5 g below the gravitational level to 4.9 m/s^2^ during down stroke. At 30 Hz, such WBV is equivalent to planet Earth expanding its mass by 50 % and then immediately shrinking it by 50 % every 0.033 s. Obviously, this condition has never occurred in Earth’s 4.54-billion-year history. Muscular activity is what typically causes such cyclic strains in bone. In other words, WBV may “fool” the bones of the body into believing that strenuous exercise is occurring, without the muscles actually having to do the work.

These data from the db/db mouse model are compelling and strongly indicate that WBV has beneficial effects on glucose metabolism in young adult type 2 diabetic mice. The reason for improved glycemic status in this study is not clear. We speculate osteocyte-dependent process as a likely contributor. Lee et al. first reported that skeleton regulates the insulin/glucose axis and energy metabolism (Lee et al. 2007). The Bone behaves as an endocrine organ by secreting osteocalcin, which leads to lower blood glucose and increase β-cell mass, insulin secretion and sensitivity. Thus, osteocalcin was considered as a treatment for T2DM (Ferron et al. 2012). Recent clinical reports have confirmed that patients with T2DM, indeed, have lower osteocalcin levels compared to normal controls (Kanazawa et al. 2009). Moreover, Fernández-Real et al. proposed that osteocalcin represents this missing link in the exercise-induced improvement in insulin sensitivity, and exercise may stimulate increased secretion of osteocalcin by the bone (Fernandez-Real et al. 2009). Meanwhile, it has been shown that WBV can potentiate bone’s anabolic responsiveness by biasing the differentiation and proliferation of mesenchymal stem cells in the marrow towards osteoblastogenesis (Luu et al. 2009). And osteocalcin, one of the very few osteoblast-specific proteins, comes from mature osteoblasts. Importantly, recently published data confer a newer model of pathogenesis for T2DM, postulating a state of low-grade inflammation preceding the acquisition of insulin intolerance; Zeng et al. (2012) describe a contributory role that an imbalance of pro- and anti-inflammatory T-cells may play in the development of T2DM. In this study we were able to show a reduced pro-inflammatory profile and less cytotoxicity under WBV condition which theoretically could be related to increased osteocalcin levels. Although osteocalcin levels were not measured in this study, clinical reports have corroborated the notion that patients with T2DM indeed have lower osteocalcin levels compared to the normal controls (Zhou et al. 2009). In a large study of 4047 men 70 years or older, Yeap et al. (2010) found that reduced total serum osteocalcin was inversely related to waist circumference, serum glucose level, serum triglyceride level, metabolic syndrome, and insulin resistance. It is thus tempting to speculate that WBV-induced euglycemic effects reflect an osteocyte-mediated increase in osteoblast release of osteocalcin, with consequently reduced inflammatory profile and cytotoxicity, ultimately leading to better insulin sensitivity. Importantly, in our experiment HbA1c levels were reduced in vibrated db/db group, and our metabolic cage study is supportive of improved glycemic status. This contention is supported by the observation of reduction in fluid intake, urine excretion output and improved urinary concentrating ability. These are relevant results, despite the small sample sizes, as indicated by the large Effective Size Indices. Accordingly, lower glucose levels may have led to a decrease in water intake, less urine output and increase in urine osmolality.

Consistent with the improvement of glycemic status, WBV exerted beneficial effects on indices of inflammation and cell injury. This contention is supported by the reduction in IL-17 positive cells but increased in Foxp3 positive cells as well as marked reduction in cells positive for gammaH2AX in peripheral blood of db/db, but not db/m, mice subjected to WBV. In our previous investigation of diabetic nephropathy, we showed marked increase in immunostaining of gammaH2AX in renal tissue of db/db than db/m mice (Mozaffari et al. 2012). Further, renal cells prepared from kidneys of db/db mice showed marked increase in percent of IL-17 positive cells compared to their db/m control (Baban et al. 2013b). In light of results of the present study, subsequent studies should establish whether WBV beneficially influences indices of inflammation and cell injury in target organs, such as the kidney, which are adversely impacted by diabetes.

In conclusion, the results of present study indicate that WBV improves glycemic status in association with improvement in indices of inflammation and cell injury in db/db mice, a very relevant animal model of T2DM. Thus, beneficial impact of WBV should be further explored in both pre-clinical and clinical studies especially in overweight or obese young adults with pre-diabetes or type 2 diabetes.
